# Anti-α4 Antibody Treatment Blocks Virus Traffic to the Brain and Gut Early, and Stabilizes CNS Injury Late in Infection

**DOI:** 10.1371/journal.ppat.1004533

**Published:** 2014-12-11

**Authors:** Jennifer H. Campbell, Eva-Maria Ratai, Patrick Autissier, David J. Nolan, Samantha Tse, Andrew D. Miller, R. Gilberto González, Marco Salemi, Tricia H. Burdo, Kenneth C. Williams

**Affiliations:** 1 Department of Biology, Boston College, Chestnut Hill, Massachusetts, United States of America; 2 Department of Radiology, Harvard Medical School, Boston, Massachusetts, United States of America; 3 Department of Neuroscience, Massachusetts General Hospital, Boston, Massachusetts, United States of America; 4 Department of Pathology, Immunology and Laboratory Medicine, University of Florida College of Medicine, Gainesville, Florida, United States of America; 5 Department of Biomedical Sciences, Section of Anatomic Pathology, College of Veterinary Medicine, Cornell University, Ithaca, New York, United States of America; Vaccine Research Center, United States of America

## Abstract

Four SIV-infected monkeys with high plasma virus and CNS injury were treated with an anti-α4 blocking antibody (natalizumab) once a week for three weeks beginning on 28 days post-infection (late). Infection in the brain and gut were quantified, and neuronal injury in the CNS was assessed by MR spectroscopy, and compared to controls with AIDS and SIV encephalitis. Treatment resulted in stabilization of ongoing neuronal injury (NAA/Cr by 1H MRS), and decreased numbers of monocytes/macrophages and productive infection (SIV p28^+^, RNA^+^) in brain and gut. Antibody treatment of six SIV infected monkeys at the time of infection (early) for 3 weeks blocked monocyte/macrophage traffic and infection in the CNS, and significantly decreased leukocyte traffic and infection in the gut. SIV – RNA and p28 was absent in the CNS and the gut. SIV DNA was undetectable in brains of five of six early treated macaques, but proviral DNA in guts of treated and control animals was equivalent. Early treated animals had low-to-no plasma LPS and sCD163. These results support the notion that monocyte/macrophage traffic late in infection drives neuronal injury and maintains CNS viral reservoirs and lesions. Leukocyte traffic early in infection seeds the CNS with virus and contributes to productive infection in the gut. Leukocyte traffic early contributes to gut pathology, bacterial translocation, and activation of innate immunity.

## Author Summary

To determine whether ongoing cell traffic is required for SIV-associated tissue damage, we blocked monocyte and T lymphocyte traffic to the brain and gut during a) ongoing infection or, b) at the time of infection. When animals were treated at four weeks post infection (late), once significant neuronal injury and accumulation of infected macrophages had already occurred, neuronal injury was stabilized, and CNS infection and the number of CNS lesions decreased. In the gut, there were significantly fewer productively infected cells and decreased inflammatory macrophages post treatment. Treatment at the time of infection (early) blocked infection of the CNS (SIV –DNA, RNA, or protein) and macrophage accumulation. In the gut, treatment at the time of infection blocked productive infection (SIV –RNA and protein) but not SIV –DNA. Interestingly, with treatment at the time of infection, there was no evidence of microbial translocation or elevated sCD163 in plasma, demonstrating that leukocyte traffic early plays a role in damage to gut tissues. Overall, these data point to the role of monocyte traffic and possibly lymphocytes to the CNS and leukocyte traffic to the gut to establish and maintain viral reservoirs. They underscore the role of monocyte/macrophage traffic and accumulation in the CNS for neuronal injury and maintenance of CNS lesions.

## Introduction

The importance of monocyte/macrophages as a critical cell type bringing human immunodeficiency virus (HIV) to the central nervous system (CNS) is often assumed [1], [2], but has not been directly investigated. Similarly, the function of leukocytes seeding the gut early during infection has not been directly assessed. HIV infection of the CNS is associated with compromised motor, behavioral, and cognitive functioning, collectively referred to as HIV-associated neurocognitive disorders (HAND) [3]. Neuropathologic correlates of these clinical conditions include accumulation of perivascular macrophages, microglial activation, decreased synaptic/dendritic densities, neuronal damage and loss [4]. Combination antiretroviral therapies (cART) restore peripheral immune function and control viral replication, however effective cART does not prevent the formation of a CNS viral reservoir early in infection [5]. Consequently, neuroinflammation remains and neurologic impairment affects the majority of HIV-infected individuals [6], [7]. Gut-associated lymphoid tissues (GALT) are another important reservoir of HIV RNA and DNA that is established during acute infection and persists despite long-term effective therapy [8], [9].

SIV infection in rhesus macaques results in a disease course similar to HIV-infected humans in the pre-ART era [10]. Experiments in SIV-infected rhesus macaques have provided important insights into the role of innate and adaptive immune cell types in viral persistence and maintenance of tissue reservoirs [11]. SIVmac251 infection with CD8 lymphocyte depletion results in uncontrolled plasma viremia during the first two weeks of infection and rapid progression to AIDS. This rapid and predictable progression to AIDS also allows for therapeutic treatment studies in monkeys because we achieve >85% incidence of AIDS and SIV encephalitis (SIVE) within months of infection compared to approximately 25% of non-depleted animals developing SIVE [11]. Similar to HIV infection in humans, virus is detected very early in the CNS, within perivascular macrophage cuffs. But in the rapid monkey model CNS pathology occurs more quickly, and histopathology is more severe with several fold more monocyte/macrophages accumulating early (21 days post infection), productive infection is easily detectable, and multi-nucleated giant cells (MNGC) are present. Within the CNS of HIV-infected humans and SIV-infected monkeys early, and terminally with AIDS, CD4+ T lymphocytes are rare, and not usually detected.

Early after exposure to HIV and SIV, virions and infected cells enter the gut and infect resident CD4+ T lymphocytes. These cells harbor virus and propagate infection, resulting in CD4+ T cell loss within days [12], [13]. With CD4+ T cell depletion, there is expansion of activated immune cells and virus in blood that can infect draining lymph nodes, brain, and other tissues [14]. CD4+ T cell apoptosis during acute HIV and SIV infection is thought to contribute to aberrant immune activation and translocation of microbial products, which can cause increased trafficking of monocytes into the CNS. It is postulated that this is closely linked to the development of HAND and SIVE [15], [16].

Similar to the gut, SIV and HIV are found in the CNS as early as 3 [17], [18] and consistently by 14 days post infection (dpi) [5], [19], and occur concurrently with accumulation of perivascular macrophages, some of which are infected [20], [21]. Although neurons are not infected, neuronal damage is evident even during the acute phase of infection [11], [22], [23]. 1H MRS is a sensitive method of non-invasively measuring neuronal injury by decreased levels of neuronal metabolites N-acetylaspartate+N-acetylaspartylglutamate (collectively NAA). Neuronal injury (decreased NAA/Cr) correlates with the expansion of activated monocytes in the periphery, indicating that neuroinvasion, likely through entry of activated or infected monocytes into the brain, is required for CNS pathogenesis [24]. We have previously shown with non-CD8 depleted SIV infected animals, decreased NAA/Cr ratios with neuronal injury that then reverse when inflammation subsides [25]. In contrast with CD8 depletion and SIV infection there is a steep and drastic decrease in NAA/Cr [26]. Using cART [27] and more recently minocycline [28] with ongoing infection and neuronal injury, we have reported a reversal of decreased NAA/Cr consistent with recovery of neuronal injury or lack of further injury. A decrease in peripheral activation of monocytes correlated with a reversal of decreased NAA/Cr [24]. Using BrdU, we have shown that the magnitude of blood monocyte expansion as early as 8 dpi is highly predictive of the rate of disease progression and severity of CNS neuropathology [29]. It is widely considered that monocyte/macrophage traffic and accumulation in CNS drives neuronal injury, though no study has tested whether directly blocking such traffic affects neuronal injury, or blocks CNS infection.

In this study, we used the anti-α4 antibody natalizumab (Biogen Idec), which selectively binds the α4 subunit of α4β1 and α4β7 integrins, blocking the interaction between α4 and its' ligands [30]. Natalizumab prevents accumulation of leukocytes (B cells, T lymphocytes, and monocyte/macrophages) in the CNS of patients with relapsing-remitting Multiple Sclerosis [31] and small intestine of patients with Crohn's disease [32], but does not affect normal leukocyte traffic through lymph nodes in humans [33], [34] or monkeys [35]. Increased expression of α4 on leukocytes and endothelial expression of VCAM-1 are critical for the migration of monocytes and T cells during both HIV and SIV infection [36]–[38], indicating that this interaction is likely involved in leukocyte migration across the brain and intestinal barriers with immune activation occurring early in disease. In this study, we used natalizumab primarily to assess the requirement of leukocyte trafficking on SIV neuropathogenesis, and secondarily to assess the impact of SIV pathogenesis in the gut. To examine the requirement of leukocytes for neuronal injury and maintenance of viral reservoirs, macaques were treated later in infection (late; 28, 34, and 41 dpi; n = 4) and compared to SIV infected non-treated controls (n = 4), all sacrificed when they developed AIDS (49 to 62 dpi). To determine if leukocyte traffic is responsible for seeding and/or maintaining viral infection of the brain and gut, animals received natalizumab at the time of infection (early; 0, 7, and 14 dpi; n = 6) and were compared to untreated controls (n = 3), all sacrificed on 22 dpi. In animals treated late, we found decreased accumulation of SIV-infected monocyte/macrophages in the CNS and stabilization of neuronal injury. Early natalizumab treatment prevented macrophage traffic and infection in the CNS, and decreased the number of productively infected cells in the gut. Overall, these data underscore the requirement of monocyte/macrophage traffic for neuronal injury and maintenance of the CNS lesions, and indicate that early leukocyte traffic is critical for seeding the CNS and contributes to seeding of gut with virus.

## Results

**Figure 1:**
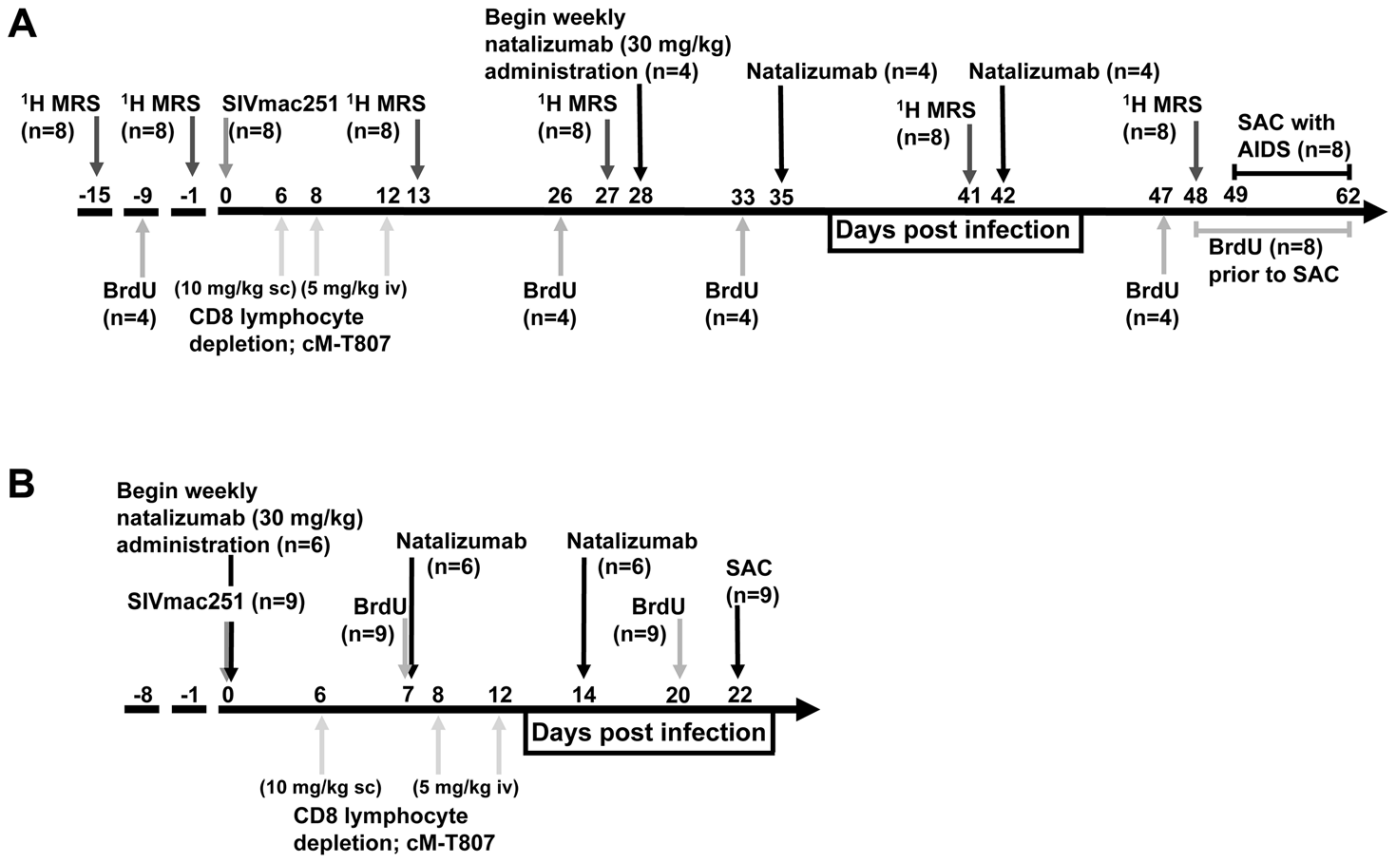
Schematic representation of study design and experimental procedures. (A) Eight rhesus macaques were infected with SIVmac251 and CD8 T lymphocyte depleted on 6, 8 and 12 dpi. In all animals, CNS changes were monitored by 1H MRS prior to infection (−15, −9 dpi) and biweekly throughout infection (13, 26, 41, 48 dpi). Four macaques were treated with natalizumab weekly for three weeks (late; 28, 35, and 41 dpi) once significant neuronal injury was observed by 1H MRS. Two untreated and two natalizumab treated macaques received BrdU throughout the experiment (−9 dpi, 26 dpi, and 24 hours prior to sacrifice) to determine the total number of cells trafficking into tissues. Two untreated and two treated macaques received BrdU on 33 dpi and 24 hours prior to sacrifice to compare the number of cells trafficking into tissues with and without natalizumab. All macaques were sacrificed upon progression to AIDS between 49–62 dpi. (B) Nine rhesus macaques were infected with SIVmac251 (0 dpi) and CD8 T lymphocyte depleted on 6, 8, and 12 dpi. Six macaques received weekly natalizumab infusions beginning at the time of infection (0 dpi), and on 7 and 14 dpi (early). Animals received BrdU on 7 and 20 dpi to monitor the total number of cells trafficking into tissues and were all sacrificed at 22 dpi.

In these experiments, two groups of animals were used: a cohort treated 28 days after infection (termed late; Fig. 1A) and a cohort treated at the time of infection (termed early; Fig. 1B). Treatment later in infection (28 dpi) was used to determine the contribution of leukocytes in maintenance of SIV reservoirs and CNS injury. Early treatment (0 dpi) was used to determine the role of leukocytes in seeding CNS and gut. A total of seventeen rhesus macaques were SIVmac251 infected and CD8 lymphocyte depleted on 6, 8, and 12 dpi. In the late group, to determine whether continuous leukocyte traffic is required for neuronal injury, changes in brain metabolites were measured by 1H MRS biweekly, and natalizumab treatment began after four weeks of infection (n = 4 natalizumab treated, n = 4 non-treated controls; Fig. 1A). To measure the total number of monocyte/macrophages trafficking out of the bone marrow, through the blood, and into tissues, BrdU was administered to n = 2 late natalizumab treated and n = 2 non-treated controls at −9 dpi, 26 dpi, and 24 hours prior to necropsy. In the other n = 2 late natalizumab and n = 2 control animals, BrdU was administered at 33 dpi and 24 hours prior to sacrifice, in order to monitor monocyte/macrophage trafficking once natalizumab treatment had begun. All animals in the late cohort (n = 4 natalizumab treated, n = 4 controls) were sacrificed with progression to AIDS (49 to 62 dpi) (Fig. 1A). In the early group, to determine whether leukocyte traffic is necessary for seeding viral reservoirs with SIV, natalizumab treatment was started at the time of infection (Fig. 1B). N = 6 early natalizumab treated and n = 3 non-treated control macaques were given BrdU on 6 and 20 dpi, prior to sacrifice on 22 dpi (Fig. 1B).

### Natalizumab treatment with ongoing infection stabilizes neuronal injury

**Figure 2:**
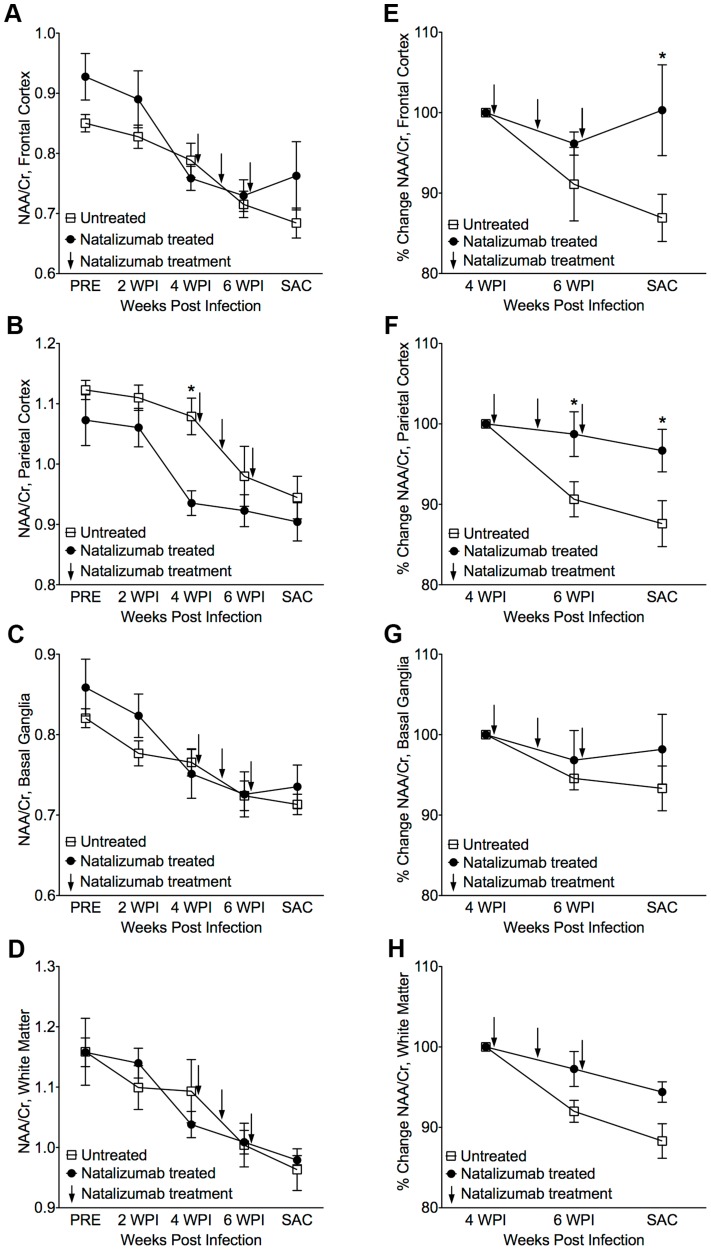
Stabilization of neuronal injury in SIV-infected animals following natalizumab treatment. (A) A decreased NAA/Cr ratio in frontal cortex (FC), (B) parietal cortex (PC), (C) basal ganglia (BG), and (D) white matter (WM), was observed in untreated and natalizumab treated animals by four weeks post infection (WPI). Decreased NAA/Cr stabilized with natalizumab treatment (indicated by arrows at 28, 35, and 42 dpi) in the FC (E), PC (F), BG (G), and WM (**H**). Each point represents the mean ± SEM. P≤0.05* using Holm-Šídák post-tests following significant repeated measures ANOVA.

The eight SIV-infected macaques in the later cohort (n = 4 natalizumab treated, n = 4 non-treated) developed AIDS. One of the four natalizumab treated animals and two of four untreated macaques developed SIVE, defined by productive viral replication, the presence of MNGC, and macrophage accumulation in the CNS. Plasma viral loads in all animals remained elevated regardless of treatment and there were no differences in viral loads between control and experimental animals (Figure S1). Interestingly, CSF viral load was elevated in early in animals that were treated at the time of infection. We assessed the requirement of continuous monocyte/macrophage traffic for neuronal injury and maintenance of CNS reservoirs with three weekly natalizumab treatments (30 mg/kg) beginning on 28 dpi, when significant neuronal damage had already occurred [22], [23]. Neuronal injury (decreased NAA/Cr) was measured in frontal cortex (FC), parietal cortex (PC), basal ganglia (BG), and white matter semiovale (WM) of the four natalizumab treated and four untreated macaques by MR spectroscopy biweekly (Fig. 2). The mean NAA/Cr ratio declined from pre-infection to 4 weeks post infection (wpi) in FC (−13%, p = 0.0028), PC (−8.3%, p = 0.0016), BG (−9.7%, p = 0.008), and WM (−8%, p = 0.036) of all animals (Fig. 2A–D), consistent with neuronal damage as previously reported [23], [26], [27]. Following natalizumab treatment, NAA/Cr decreases stabilized in the FC (+0.5%, p = 0.892), PC (−3.3%, p = 0.596), BG (−2.1%, p = 0.757) and WM (−5.7%, p = 0.046). In contrast, SIV infected, non-treated animals had continued reductions of NAA/Cr in the FC (−13.2%, p = 0.016), PC (−12.5%, p = 0.0008), and WM (−11.9%, p = 0.0001), and a trend towards decline in the BG (−6.8%, p = 0.13) as previously demonstrated (Fig. 2E–H) [26]–[28]. When comparing the NAA/Cr slopes following natalizumab treatment, there was a significant difference between groups in the PC (untreated r^2^ = 0.76, treated r^2^ = 0.21; p = 0.038) and WM (untreated r^2^ = 0.83, treated r^2^ = 0.76; p = 0.031), but not the FC (untreated r^2^ = 0.77, treated r^2^ = 0.0005; p = 0.057) and BG (untreated r^2^ = 0.49, treated r^2^ = 0.28; p = 0.362). Along with NAA/Cr, changes in Myo-inositol (MI) and Choline (Cho) were monitored throughout infection, with no significant differences in MI/Cr or Cho/Cr between treatment groups at any point during the study.

### Accumulation of leukocytes in the blood of natalizumab treated animals

Relative to control animals, there were increased numbers of leukocytes and leukocyte precursors in the blood with natalizumab, which is likely due to their inability to traffic to parenchymal, non lymphoid tissues (Table 1). In late treated macaques, we found elevated numbers of CD4^+^ lymphocytes (control: 450 cells/µl, treated: 1444 cells/µl), CD14^+^ monocytes (control: 260 cells/µl, treated: 879 cells/µl) CD14^l^°CD16^+^ monocytes (control: 15 cells/µl, treated: 48 cells/µl), CD20^+^ B cells (control: 464 cells/µl, treated: 1140 cells/µl), and CD34^+^ hematopoetic stem cells (HSCs) (control: 1.9 cells/µl, treated: 21 cells/µl). With early natalizumab treatment, there were elevated numbers of circulating CD4^+^ lymphocytes (control: 536 cells/µl, treated: 903 cells/µl), CD14^+^ monocytes (control: 355 cells/µl, treated: 725 cells/µl), CD14^+^CD16^−^ monocytes (control: 260 cells/µl, treated: 467 cells/µl), CD14^+^CD16^+^ monocytes (control: 55 cells/µl, treated: 123 cells/µl), CD14^l^°CD16^+^ monocytes (control: 17 cells/µl, treated: 96 cells/µl), CD20^+^ B cells (control: 91 cells/µl, treated: 415 cells/µl), and CD34^+^ HSCs (control: 1 cell/µl, treated 30 cells/µl). These data indicate that α4 blockade with natalizumab prevented cell trafficking, as previously described in humans [31], [39], [40] and monkeys [35]. Although there was accumulation of all immune cell subsets in the blood, this increase only affected the percentage of CD14^l^°CD16^+^ monocytes, B cells, and HSCs in late natalizumab treated animals, and the proportions of CD14^+^CD16^+^ and CD14^l^°CD16^+^ monocytes, CD20^+^ B cells, and CD34^+^ HSCs with early natalizumab treatment (Table 1). All control and natalizumab treated animals remained CD8 lymphocyte depleted throughout the study (Table 1). In tissues we found no CD3^+^CD8^+^ lymphocytes in the CNS of early and late natalizumab treated or control animals, nor were there CD3^+^CD8^+^ lymphocytes in lymph nodes. This is consistent with these animals being persistently CD8 lymphocyte depleted as we have previously described [11], [24], [27], [29], [41]. Similarly, we did not find CD3^+^CD8^−^ lymphocytes (consistent with CD3^+^CD4^+^ cells) in the CNS. In gut regions sampled, we did not find CD3^+^CD8^+^ lymphocytes, but did find CD3^+^CD8^−^ lymphocytes (see below) that represent CD4^+^ lymphocytes and/or NK cells. Together, these data are consistent with blocking leukocyte traffic to the CNS and gut with natalizumab.

**Table 1:** The percentages and absolute cell numbers of lymphocytes, monocytes, B cells, and hematopoietic stem cells in late and early SIV-infected animals.

	Late						Early					
	Absolute Cell Number (cells/ul)			% of Total Leukocytes			Absolute Cell Number (cells/ul)			% of Total Leukocytes		
	27 dpi	49 dpi		27 dpi	49 dpi		0 dpi	22 dpi		0 dpi	22 dpi	
	All Animals	Control	NZ Treated	All Animals	Control	NZ Treated	All Animals	Control	NZ Treated	All Animals	Control	NZ Treated
	(n = 8)	(n = 4)	(n = 4)	(n = 8)	(n = 4)	(n = 4)	(n = 9)	(n = 3)	(n = 6)	(n = 9)	(n = 3)	(n = 6)
**CD4+ Lymphocytes**	684 (77)	450 (68)	1444 (182)	11.5 (2)	12 (4)	20 (2.9)	890 (82)	536 (244)	903 (134)	12.7 (1.1)	10.8 (2.8)	14.7 (3)
**CD8+ Lymphocytes**	1.7 (0.5)	1.2 (0.7)	1.2 (0.6)	0.3 (0.2)	0.03 (0.01)	0.4 (0.05)	3.81 (4.8)	5.4 (1.4)	2.2 (0.3)	7.6 (1.5)	0.16 (0.05)	0.42 (0.08)
**CD14+ Monocytes**	424 (61)	260 (49)	879 (239)	7 (0.9)	6 (1)	10 (2)	243 (38)	355 (41)	725 (183)	3 (0.6)	8 (0.8)	9 (2)
**CD14+ CD16−**	295 (56)	328 (135)	364 (143)	6.4 (0.5)	4.5 (1)	6 (0.3)	219 (36)	260 (76)	467 (160)	72 (5)	64 (10)	68 (5)
**CD14+ CD16+**	98 (21)	55 (22)	74 (14)	1.9 (0.4)	2 (1)	2 (0.3)	23 (4)	55 (13)	123 (36)	6 (1.6)	1.4 (0.4)	17 (4)
**CD14lo CD16+**	7 (1.5)	15 (5)	48 (16)	1.4 (0.4)	1.1 (0.8)	9 (2.4)	16 (3)	17 (9)	99 (16)	4 (1.5)	0.5 (0.2)	10 (3)
**CD20+ B Cells**	221 (69)	464 (252)	1140 (369)	9 (1)	14 (3)	32 (5)	583 (120)	91 (22)	415 (99)	19 (3)	8 (1)	21 (3)
**CD34+ HSCs**	2.2 (1)	1.9 (0.9)	21 (4.3)	1 (0.3)	0.04 (0.02)	7 (1.7)	2 (0.5)	1 (0.2)	30 (7)	0.7 (0.2)	0.5 (0.4)	25 (4)

Note- Data represent the means of all animals prior to and following natalizumab treatment with the SEM in brackets.

NZ = natalizumab. HSC = Hematopoietic Stem Cell.

### Late natalizumab treatment suppresses the traffic and accumulation of SIV infected monocyte/macrophages in the brain

**Figure 3:**
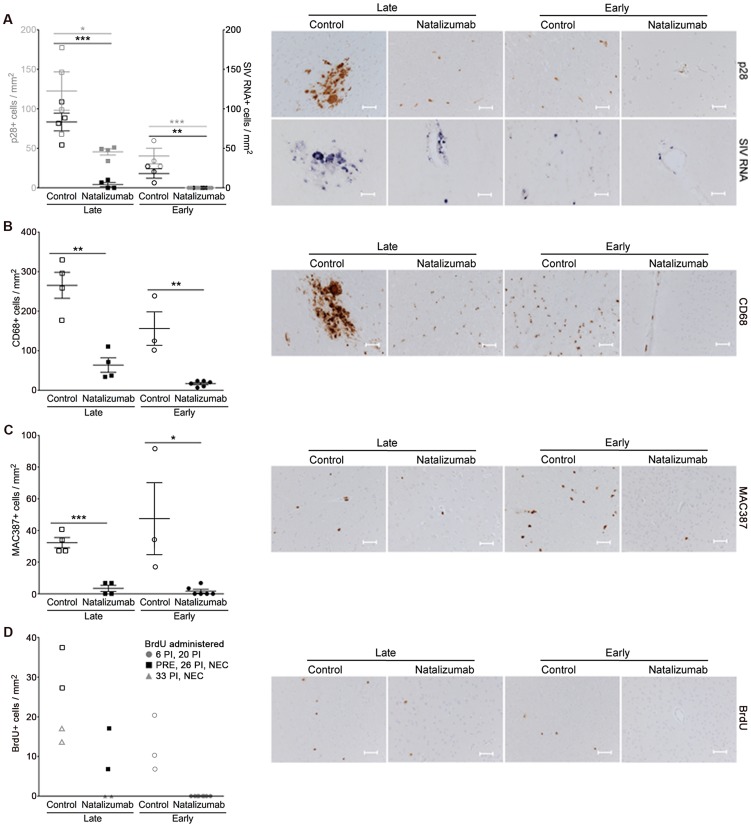
Natalizumab treatment blocks the traffic and accumulation of SIV infected monocyte/macrophages in the brain. (A) Natalizumab treatment on 28 dpi (late), resulted in scattered SIV p28^+^ and RNA^+^ cells in the CNS (frontal cortex, parietal cortex, occipital cortex, brainstem) relative to controls, all sacrificed with progression to AIDS. Natalizumab at the time of infection (early) prevented the traffic of SIV p28^+^ and RNA^+^ cells into the parenchyma, while several SIV p28^+^ and RNA^+^ cells were evident in tissues of untreated controls that were all sacrificed on 22 dpi. (B) Late natalizumab treatment resulted in decreased numbers of CD68^+^ macrophages in the brain relative to controls. Numbers of CD68^+^ macrophages were significantly reduced in the brains of early natalizumab treated animals compared to matched controls. (C) Fewer MAC387^+^ cells were observed in late natalizumab treated macaques compared to non-treated animals. Significantly less MAC387^+^ monocytes were detected in early treated macaques than in untreated controls. (D) To determine the timing of monocyte/macrophage egress into the CNS, animals were administered BrdU at various time points. In animals given BrdU after late natalizumab treatment had begun (33 dpi, 24-hours prior to necropsy), there were no BrdU^+^ monocyte/macrophages any brain region examined. Lower numbers of BrdU^+^ cells were detected in natalizumab treated than non-treated animals that all received BrdU throughout infection (−9 dpi, 26 dpi, and 24-hours prior to necropsy). No recently recruited BrdU^+^ monocyte/macrophages were found in the parenchyma of animals treated with early natalizumab. Scale bars: 50 microns. P values calculated using unpaired t tests. P≤0.05*, p≤0.01**, p≤0.001 ***.

In all brain regions examined (frontal cortex, parietal cortex, occipital cortex, brainstem), numbers of SIV p28^+^ and RNA^+^ cells were markedly lower in late natalizumab treated versus untreated animals (p28^+^ p = 0.0202, RNA^+^ p = 0.0005; Fig. 3A). There were significantly fewer activated CD68^+^ resident macrophages (p = 0.0017; Fig. 3B) and recently infiltrating MAC387^+^ monocytes (p = 0.0003; Fig. 3C) in late treated macaques. We have previously observed significant numbers of BrdU^+^ macrophages in the CNS of animals receiving BrdU even 24-hours prior to sacrifice [29], yet no BrdU^+^ macrophages were found in brains of animals that received BrdU after natalizumab treatment began (33 dpi, 24-hours prior to necropsy) (Fig. 3D). In animals that received BrdU throughout infection (−9 dpi, 26 dpi, and 24-hours prior to necropsy), we found lower numbers of BrdU^+^ cells in natalizumab treated animals versus controls (Fig. 3D). These data demonstrate that natalizumab treatment with ongoing infection blocks monocyte/macrophage traffic, reduces the CNS reservoir of productively infected monocyte/macrophages, and stabilizes neuronal injury.

### Reduced accumulation of T lymphocytes, monocyte/macrophages, and productively SIV infected cells in the gut with late natalizumab treatment

**Figure 4:**
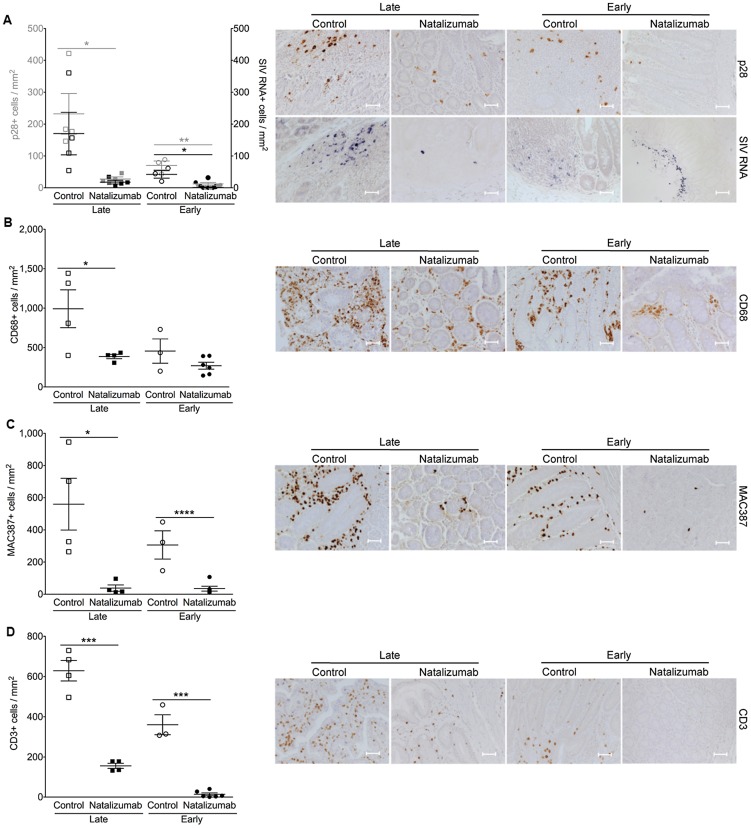
Natalizumab reduces accumulation of productively infected cells, CD3^+^ T cells, and MAC387^+^ monocytes to gut. (A) There are significantly fewer SIV p28^+^ infected cells in the duodenum, jejunum, and colon tissues from late natalizumab treated animals compared to untreated animals. The numbers of SIV RNA^+^ cells were similar between both groups. Less SIV p28^+^ and RNA^+^ cells were detected in guts of early natalizumab treated macaques compared to controls. (B) Untreated animals had significantly higher numbers of CD68^+^ macrophages than late natalizumab treated animals. There were comparable numbers of CD68^+^ macrophages in gut tissues from early natalizumab treated and non-treated animals. (C) Untreated controls had significantly higher numbers of MAC387^+^ cells in the intestine than macaques starting natalizumab late in infection. Natalizumab treatment early reduced the number of infiltrating MAC387^+^ monocytes compared to controls. (D) There were fewer CD3^+^ T lymphocytes in the gut of late and early natalizumab treated animals compared to controls. Lines and error bars indicate the mean ± SEM for each treatment group. Scale bars: 50 microns. P values calculated using unpaired t tests. P≤0.05*, p≤0.01**, p≤0.001 ***, p≤0.0001 ****.

In the gut (duodenum, jejunum, colon), there were fewer SIV p28^+^ cells in treated animals (p = 0.0187), but no difference in the number of RNA^+^ cells observed between late natalizumab treated animals and untreated controls (Fig. 4A). There were lower numbers of CD68^+^ macrophages (p = 0.0460; Fig. 4B), MAC387^+^ monocytes (p = 0.0182; Fig. 4C), and CD3^+^ T lymphocytes (p = 0.0001; Fig. 4D) in the guts of natalizumab treated macaques, indicating that late treatment was not sufficient to stop viral infection that has already occurred, but did reduce subsequent traffic of lymphocytes and monocyte/macrophages.

### Early natalizumab treatment blocks traffic and accumulation of SIV infected monocyte/macrophages in the brain and gut, and bacterial translocation

**Figure 5:**
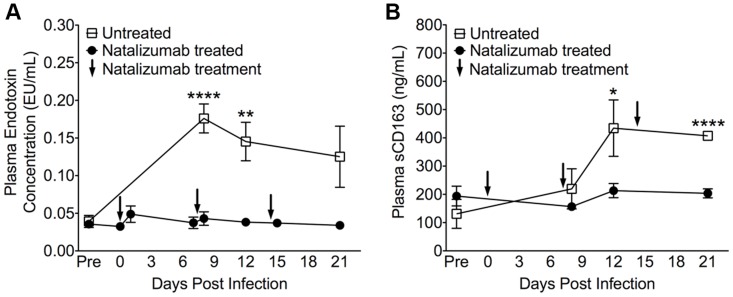
Reduced LPS and sCD163 levels in plasma with early natalizumab treatment. (A) There were high levels of LPS in plasma on 8, 12, and 21 dpi in control macaques, with significant differences between natalizumab treated (represented by arrows at 0, 7, and 14 dpi) and untreated groups on days 8 and 12 pi. (B) With natalizumab treatment, soluble CD163 (sCD163) remained stable throughout the study, whereas sCD163 increased significantly in untreated animals at both 12 and 21 days post infection. Endotoxin (LPS) and sCD163 levels were determined in duplicate. Each point represents the mean ± SEM and p values were calculated using unpaired t tests. P≤0.05*, p≤0.01**, p≤0.0001****.

Next we sought to determine whether weekly natalizumab treatment at the time of infection (early) blocks viral seeding of the CNS and gut. Relative to untreated controls (n = 3) that were also sacrificed at 22 dpi, there were fewer SIV p28^+^ (p = 0.0004) and RNA^+^ (p = 0.0024) cells (Fig. 3A), and CD68^+^ macrophages (p = 0.0016; Fig. 3B) in the CNS of early natalizumab treated animals (n = 6). When present, SIV p28^+^ and RNA^+^ cells were primarily found in vessels outside the parenchyma. Numbers of MAC387^+^ cells were lower in brains of early treated macaques (p = 0.0179; Fig. 3C), and recently trafficking BrdU^+^ cells were absent (Fig. 3D). In the guts of animals receiving early natalizumab, there was a significant reduction in SIV p28^+^ (p = 0.0012) and RNA^+^ cells (p = 0.0013) (Fig. 4A). There were similar numbers of CD68^+^ macrophages in early treated and control groups (Fig. 4B), but lower numbers of MAC387^+^ monocytes (p<0.0001; Fig. 4C) and CD3^+^ T lymphocytes (p = 0.0001; Fig. 4D) with natalizumab treatment. Interestingly, early treated macaques had significantly lower plasma LPS at 8 (p<0.0001) and 12 dpi (p = 0.0019) than untreated controls (Fig. 5A). In contrast, equivalent LPS levels were seen in late treated and non-treated animals. Early natalizumab also resulted in reduced soluble CD163 in plasma, with treated macaques exhibiting significantly lower concentrations than untreated macaques at 12 (p = 0.0488) and 21 dpi (p<0.0001) (Fig. 5B).

### Early natalizumab blocks viral seeding in brain, but not in gut and lymph nodes

**Figure 6:**
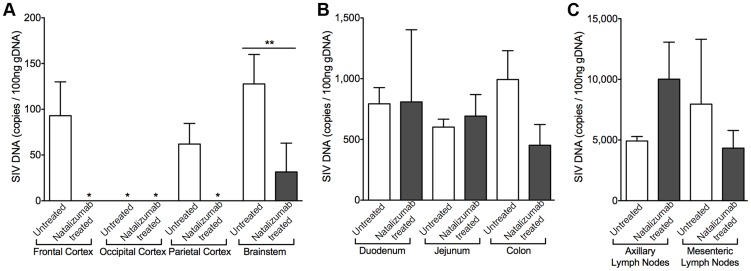
Early natalizumab treatment blocks viral DNA expression in brain, but not gut and lymph nodes. (A) SIV *gag* DNA was undetectable in all but one of the 24 brain tissue samples analyzed from animals treated with natalizumab on the day of SIV infection. (B) Similar viral DNA copy numbers were found in the duodenum and jejunum tissues of all macaques sacrificed at 22 dpi, regardless of treatment. The lowest concentrations of SIV DNA were present in the colon of natalizumab treated animals, in contrast to untreated macaques, which had much higher levels of SIV DNA in this region. (C) The number of proviral DNA copies was higher in the axillary lymph nodes of treated than in non-treated animals whereas SIV DNA was lower with natalizumab treatment in mesenteric lymph node tissue. Viral DNA copies were measured in duplicate. Each bar represents the mean ± SEM for each animal group. P values calculated using unpaired t tests. p≤0.01 **.

To determine whether natalizumab treatment on the day of SIV infection blocked latent viral infection in brain and gut, we analyzed tissues for SIV *gag* DNA using qPCR. Proviral DNA was undetectable in brains of five of six early natalizumab treated macaques (Fig. 6A). One animal had a low level of SIV DNA that was detected only in brainstem, which may be explained by brainstem trauma resulting from a CSF tap. Although natalizumab significantly reduced the number of productively infected cells in the gut, similar numbers of viral DNA copies were found in the duodenum and jejunum of natalizumab and untreated animals. SIV *gag* DNA levels were lower in colon with treatment, however this difference did not reach significance (Fig. 6B).

### Natalizumab treatment does not affect monocyte/macrophage traffic or the accumulation of productively infected macrophages in lymph nodes

**Figure 7:**
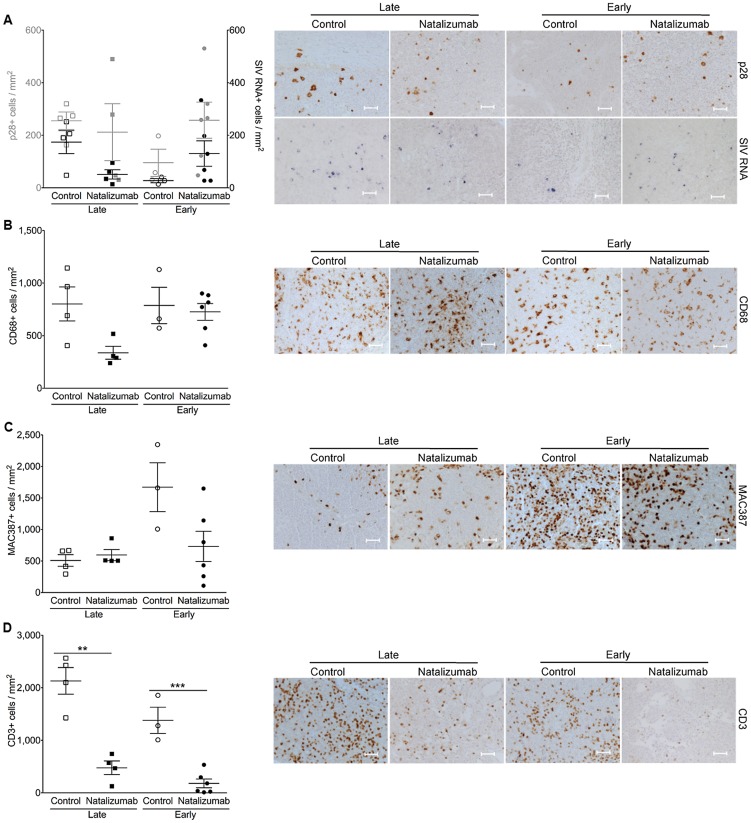
There are fewer CD3+ T cells, but not monocyte/macrophages in lymph node with natalizumab treatment. (A) There were similar numbers of p28^+^ and SIV RNA^+^ cells in the axillary lymph nodes of all of animals, regardless of treatment or time of sacrifice. (B) Comparable levels of activated resident CD68^+^ macrophages were detected in all macaques. (C) The numbers of MAC387^+^ cells were similar in late and early treated versus non-treated macaques. (D) There was a reduction in CD3^+^ T lymphocytes from tissues of late and early natalizumab treated macaques relative to untreated controls. Each point represents the mean number of positive cells in the three tissue regions examined from a single animal. Lines and error bars indicate the mean ± SEM for each treatment group. Scale bars: 50 microns. P values calculated using unpaired t tests. P≤0.05*, p≤0.01 **.

There was elevated SIV provirus in axillary lymph nodes and similar levels of SIV DNA in mesenteric lymph nodes of early natalizumab treated relative to untreated controls (Fig. 6C), probably reflecting differing degrees of α4β1 and α4β7 utilization in these different compartments. This was not surprising, as comparable numbers of SIV p28^+^ and RNA^+^ infected cells were detected in lymph nodes from treated and untreated animals in both late and early cohorts (Fig. 7A). Natalizumab treated animals had fewer CD3^+^ T lymphocytes in lymph nodes than matched controls (Late p = 0.0011, Early p = 0.0006; Fig. 7D), yet similar numbers of CD68^+^ (Fig. 7B) and MAC387^+^ monocytes (Fig. 7C) were observed in all animals, suggesting that natalizumab did not affect immune recirculation in lymph nodes.

## Discussion

While it has been suggested that monocyte/macrophage traffic drives CNS infection and neuron damage, this has not been demonstrated experimentally. Here, we examined whether continuous neuronal injury with HIV and SIV infection depends on monocyte/macrophage traffic, and if cell trafficking to CNS and gut is required for viral seeding. NAA/Cr was monitored throughout infection in four SIV-infected rhesus macaques treated with natalizumab beginning after 28 days of infection, when significant neuronal injury had already occurred. We have previously shown these decreases to correlate with increased monocyte/macrophage activation, accumulation, viral infection, and neuronal injury by immunohistochemical and neuropathologic examination [11], [22], [24], [42]. Despite significant reductions in NAA/Cr, blocking cell traffic with natalizumab stabilized NAA/Cr declines, consistent with limiting further neuronal injury. Because natalizumab also blocks lymphocyte traffic, it is possible that lymphocytes might also play a role in neuronal injury, however it has been repeatedly demonstrated that there are low-to-no CD4^+^ T cells in the CNS with HIV and SIV infection [11], [43], [44], and our animals were CD8 lymphocyte depleted. Although not absolutely demonstrated, this underscores the importance of monocyte/macrophage more so than lymphocyte traffic in SIV neuropathogenesis.

To determine whether leukocyte traffic is required for initial seeding of brain and gut, animals were treated with natalizumab beginning on the day of infection. At sacrifice 22 days later, no SIV p28^+^ or RNA^+^ cells were found in the CNS, indicating that traffic of leukocytes from the periphery is necessary for initial viral dissemination in the brain. This is further supported by the absence of SIV *gag* DNA in brain tissues of five of six natalizumab treated animals. Provirus in the brainstem of the sixth macaque may be a result of a CSF tap trauma, and a lower concentration of SIV *gag* DNA was found in this animal than in brains of non-treated controls.

There were no BrdU^+^ cells in early or late natalizumab treated animals, indicating that α4 blockade was sufficient to prevent BrdU^+^ monocyte/macrophages from entering the brain. We have previously reported that the majority of BrdU^+^ cells in the CNS of SIV-infected animals are MAC387^+^ [29], [41], underscoring the role of recently recruited MAC387^+^ monocytes in active CNS inflammation [11]. The few scattered MAC387^+^ monocytes and CD68^+^ macrophages seen in brains of treated animals suggests that despite SIV infection and CD8 lymphocyte depletion, very little inflammation occurred in the CNS following natalizumab treatment. Blocking leukocyte traffic later in animals with ongoing inflammation and lesions reduced inflammation to almost undetectable levels. These observations with low numbers of SIV p28^+^ and RNA^+^ cells and rapid stabilization of NAA/Cr in the brains of late natalizumab treated animals suggest that ongoing traffic maintains not only neuronal injury, but also productive infection of the CNS.

The small intestine is a primary site for SIV infection, with interaction between the α4β7 integrin and MAdCAM-1 facilitating traffic of leukocytes [45], [46]. Natalizumab reduced numbers of CD3^+^ T lymphocytes, MAC387^+^ monocytes, and SIV p28^+^ cells relative to controls, suggesting that treatment suppressed traffic of cells responsible for early viral replication. It has previously been shown that loss of α4β7^HIGH^CD4^+^ T cells in blood is an indication of decreased numbers of CD4^+^ T cells in gut [47]. Whether we directly blocked trafficking of α4β7^HIGH^CD4^+^ T cells was not assessed. SIV DNA was detected in gut tissues of early natalizumab treated animals, however it is plausible this is non-integrated DNA, as very low numbers of SIV p28^+^ and RNA^+^ cells were observed in these tissues. Others have demonstrated that with early infection of GALT with ART given four hours after infection, there is protection against rapid depletion of CD4^+^ T cells, yet SIV RNA and DNA were detected [48]. Despite high levels of SIV provirus in the gastrointestinal tract with natalizumab treatment, productive viral infection appeared to be controlled. This is in contrast to what was seen in the CNS, which might be accounted for in part by the BBB. It is established that in the CNS, the BBB controls traffic of cells, which can be blocked by natalizumab. Therefore, blocking α4β1 and α4β7 likely has a more limited impact in gut than in the CNS. It is important to note that the majority of T cells trafficking to GALT utilize α4β7, but this is a small population in the blood [46], [49]. The viral envelope protein gp120 can bind to the α4β7 receptor expressed by leukocytes homing to the small intestine, which may not affect cell infection, but can result in activation and apoptosis of T lymphocytes by HIV and SIV [50]. This could be why we observed low numbers of CD3^+^ T cells despite similar levels of SIV DNA in the guts of natalizumab treated animals.

We did not find differences in plasma and CSF viral loads with and without natalizumab treatment (early and late). The lack of differences in plasma underscores that plasma viral load in this study is not driving tissue pathogenesis, and is more likely the result of cell-associated virus and inflammatory cells. The significance of these observations is not clear. It is unclear whether treatment late, once virus has entered the CNS, would be expected to have an effect on CSF virus since CNS parenchymal infection has already been established. The lack of differences in early treatment could be the result of virus entering the CNS as free virus, but to date this has not been convincingly demonstrated and there was no productive infection in the parenchyma. A more likely scenario is that cell-associated virus enters the CNS via the area postrema that has no BBB, and/or the choroid plexus, which has tight junctions ependymal cells, but not endothelial cells. Thus, there may be minimal receptors that are blocked with natalizumab and therefore cell traffic may not be blocked. Early inflammation and traffic of leukocytes to the choroid plexus with HIV and SIV infection and evidence of productive viral infection does occur in the choroid plexus. These observations support that the choroid plexus is a likely source of elevated virus we find in the CSF, despite natalizumab treatment. With regard to decreased traffic of cells to the gut, we found low levels of plasma LPS with early natalizumab treatment, but no difference with late treatment, suggesting that inflammation in the intestine early during infection contributes to mucosal damage and endotoxin translocation. In addition to inhibiting release of microbial products such as LPS from gut, low levels of sCD163 in plasma were also observed. Because significant reductions of chemokine/cytokine production in blood and CSF have previously been shown in natalizumab treated patients [51], this may also have contributed to an overall reduction in peripheral immune activation, as suggested by the reduced sCD163 and chemokine expression on monocyte and T lymphocyte populations in blood. We found decreases in ex vivo transmigration and adhesion of PBMCs from natalizumab treated animals, further supporting the diminished ability of cells to traffic to the brain and gut.

Similar numbers of MAC387^+^ and CD68^+^ monocyte/macrophages in lymph tissues of untreated and treated macaques suggest that natalizumab did not significantly affect traffic of these cells to lymph nodes, a finding made by others using natalizumab in monkeys [35], [52]. These same studies also demonstrated normal regulatory immune function in natalizumab treated macaques, but increased numbers of lymphocyte precursors, monocyte/macrophages, and T cells in blood. We observed a similar expansion of CD14^+^ monocytes, CD4^+^ T lymphocytes, CD34^+^ hematopoietic progenitors, and CD20^+^ B lymphocytes (Table 1), as well as a decline of CD49d (α4 integrin) expression in the periphery of all treated animals. It was surprising to observe fewer CD3^+^ T lymphocytes in lymph nodes of both groups of natalizumab treated macaques, however this may be explained by higher numbers of SIV p28^+^ and RNA^+^ cells and elevated SIV DNA copies in lymph nodes, and therefore high numbers of infected leukocytes that are susceptible to apoptosis. Several papers have shown normal lymphoid follicle function and no major differences in immune function with natalizumab treatment, as both monocytes and T cells use the interaction between leukocyte function antigen (LFA) -1 with ICAM-1 or ICAM-2 in order to traffic into high endothelial venules [35], [53], [54].

Early initiation of effective cART reduces CNS disease [55], suppresses virus to non-detectable levels, and reduces HIV transmission, however current therapies are not sufficient to eradicate viral reservoirs [56]. Furthermore, many cART therapies have low CNS penetration and do not target monocyte/macrophages that drive cardiac and CNS pathology. While we do not suggest using natalizumab long-term in HIV-infected patients, one might consider whether natalizumab treatment early, in combination with antiretroviral therapy, could stop productive infection of the brain and gut, preventing the establishment of these tissue reservoirs. While PML is a concern in patients receiving natalizumab for extended periods, all reported incidents have occurred after more than a year of antibody treatment. Additionally, patients with JC viral antibodies have received effective natalizumab treatment for 24 months without the development of PML [57]. Regardless, the experiments described here underscore the critical role of monocyte/macrophage traffic in ongoing neuronal injury, and establishment and maintenance of viral reservoirs in the CNS and intestinal tissues.

## Materials and Methods

### Animals, SIV infection, CD8 lymphocyte depletion, and viral load determination

A total of seventeen rhesus macaques (*Macaca mulatta*) were intravenously inoculated with SIVmac251 (20 ng SIV p27; a generous gift from Dr. Ronald Desrosiers, NERPC). CD8 lymphocyte depletion was achieved using cM-T807, an α-CD8 antibody that was administered subcutaneously (10 mg/kg) on day 6 post infection (pi) and intravenously (5 mg/kg) on days 8 and 12 pi [58]–[60]. Eight macaques (n = 4 late natalizumab treated, n = 4 untreated) were sacrificed at similar time points with progression to AIDS (49 to 62 dpi). Nine animals (n = 6 early natalizumab treated, n = 3 untreated) were sacrificed at 22 dpi. Plasma and CSF SIV RNA were quantified in all animals at various time points throughout infection using real-time PCR as previously described [60].

### Anti-α4 integrin (natalizumab) administration

The recombinant humanized IgG4 monoclonal anti-α4 integrin mAb (natalizumab) was kindly provided by Biogen Idec (Cambridge, MA) in a sterile concentrated solution. This antibody has specificity for the α4 subunit of α4β1 (very late activation antigen 4, VLA-4) and α4β7 integrins expressed on the surface of all leukocytes except neutrophils [61]. The rhesus macaque α4 sequence exhibits 96% homology with the human sequence (NCBI), and the anti-α4 antibody binds to the α4 subunit with affinity comparable to that in humans (K_d_ = 0.04–0.07 µg/ml) [35]. The pharmacokinetic half-life of natalizumab in humans is 11±4 days, however more than 70% of α4 integrin sites remain saturated 4 weeks after infusion and cell counts in the CSF are significantly reduced for up to 6 months [33]. The antibody was administered once weekly for three weeks beginning on the day of infection (0 dpi, n = 6) or 28 days after infection (28 dpi, n = 4). On the day of infusion 30 mg/kg of α-VLA-4 was injected into a 250 mL bag of 0.9% NaCl and administered intravenously (iv) over 30–60 minutes. We chose a high dose of natalizumab and only treated three times with one-week intervals between each treatment to avoid hypersensitivity responses by the monkeys to the humanized antibody. This regimen has previously been shown to maintain high serum levels of natalizumab throughout treatment in rhesus macaques [35]. Chemistry panels including alanine aminotransferase (ALT) and aspartate aminotransferase (AST) were examined at various time points throughout infection and remained below 100 IU/L, indicating that natalizumab treatment did not induce hepatotoxicity.

### BrdU administration

5-bromo-2′-deoxyduridine (BrdU) (Sigma) was prepared as a 30 mg/mL stock solution in 1× PBS (Ca^2+^/Mg^2+^ free; Mediatech Inc.) and given intravenously at 60 mg/kg as described previously [29]. To monitor levels of monocyte/macrophage trafficking out of the bone marrow, in blood, and into the CNS and gut, BrdU was administered prior to infection (−9 dpi), at peak infection (26 dpi), and 24 hours prior to necropsy in two macaques given natalizumab beginning on 28 dpi and two untreated control animals. In the other thirteen animals, BrdU was administered once natalizumab treatment was initiated, on days 33 and 47 post infection (n = 2 late treated, n = 2 untreated) or days 6 and 20 post infection (n = 6 early treated, n = 3 untreated).

### Flow cytometry

Flow cytometric analyses were performed as previously published [24], [62] using 100 µl samples of blood stained with the following fluorochrome-conjugated primary antibodies: anti-CD3-Alexa Fluor 700 (SP34-2), anti-CD4-PerCp-Cy5.5 (L200), anti CD8-APC (RPA-T8), anti-CD11b-Alexa Fluor 700 (1CRF44), anti-CD14-Pacific Blue (M5E2), anti-CD16-PE-Cy7 (3G8), anti-CD20-APC (2H7), anti-CD20-APC-Cy7 (L27), anti-CD25-PE (M-A251), anti-CD34-PE (563), anti-CD49d-PE-Cy5 (9F10), anti-CD95-FITC (DX2), anti-CD195-APC (3A9), and isotype control anti-IgG_1_, κ-FITC (DX2) from BD Biosciences, HLA-DR-Texas Red-PE (Immu-357; Beckman Coulter), anti-CD163-PerCp-Cy5.5 (GHI/61; Biolegend), anti-CD8-PE (DK25; Dako), anti-CD28-PE-Cy7 (CD28.2; eBioscience), anti-CD8-Qdot-655 (3B5; Invitrogen); anti-CD44v6-Biotin (VFF-7; Invitrogen), anti-CD4-Qdot-605 (19Thy-5D7; NIH Nonhuman Primate Reagent Resource), anti-CCR2-PE (48607; R&D systems), and anti-CD64-FITC (22; Trillium Diagnostics). Samples were fixed in PBS containing 2% formaldehyde, acquired on a FACSAria cell sorter (Becton-Dickinson) and analyzed with Tree Star Flow Jo version 8.7. Monocytes and lymphocytes were first selected based on size and granularity using forward scatter (FSC) area vs. side scatter (SSC) area. From this gate, doublets were excluded (FSC area vs. FSC height). Populations were further identified using negative selection and positive expression of various cell markers using 12-color flow cytometry panels. Complete blood counts were obtained using a CBC Hematology Analyzer (Hema-True, HESKA) and the absolute number of peripheral blood cell subsets was calculated by multiplying the total white blood cell count by the total percentage of each population as determined by flow cytometric analysis.

### MRI/MRS

To determine if blocking monocyte/macrophage traffic impacted neuronal injury, n = 4 rhesus macaques were treated with natalizumab beginning on 28 dpi. These animals and non-treated controls (n = 4) were scanned prior to infection (2×) and biweekly thereafter until sacrifice. For imaging, each animal was tranquilized, intubated, and monitored continuously throughout the scanning procedure as previously described [26], [28]. Briefly, MR imaging and spectroscopy were performed on a 3 Tesla whole-body imager (Magnetom TIM Trio, Siemens) with a circularly polarized transmit-receive extremity coil. First a three-plane localizer scan, used for positioning and to ensure 1H voxel reproducibility, was acquired. The 1H MRS volumes of interest (VOI) were then chosen as previously described [26], [28]. Single-voxel proton spectra were acquired from the parietal cortex (PC), frontal cortex (FC), basal ganglia (BG) and white matter semiovale (WM) using the point resolved spectroscopy sequence (PRESS) with WET [63] water suppression. Spectroscopic data were processed using LCModel software and concentrations of NAA (N-acetylaspartate+N-acetylaspartylglutamate) and creatine-containing compounds (Cr) were quantified using the unsuppressed water signal as an internal intensity reference.

### Soluble CD163 ELISA and LAL assay for LPS in plasma

Levels of sCD163 in plasma were determined using an ELISA kit, according to the manufacturer's protocol (Trillium Diagnostics) as previously described [29]. Endotoxin lipopolysaccharide (LPS) levels in heat-inactivated plasma were measured using the Limulus Amebocyte Lysate (LAL) test (Associates of Cape Cod Inc.) as previously described [29]. Samples were diluted fivefold with endotoxin-free water and heated (30 min at 65°C) to inactivate plasma components. Following incubation with LAL (30 min at 37°C) and chromogen, duplicate samples were read at 570 nm in a photometric plate reader. LPS concentrations were expressed in endotoxin units (EU), with an assay sensitivity range of 0.005 EU/mL–50 EU/mL.

### Immunohistochemistry and in situ hybridization

On the day of sacrifice, animals were anesthetized with ketamine-HCl and euthanized by intravenous pentobarbital overdose. Axillary lymph node, intestinal (duodenum, jejunum, and colon), and cerebral (brainstem, frontal cortex, parietal cortex, and occipital cortex) tissues were collected in 10% neutral buffered formalin, embedded in paraffin, and sectioned at 5 µm. For immunohistochemistry, tissue sections were deparafinized, rehydrated and incubated with blocking reagents. Mature resident monocyte/macrophage and activated microglia were assessed using anti-CD68 (KP1; Dako) and newly infiltrating monocytes were identified by the expression of myeloid/histiocyte antigen MAC387 (MAC387; Dako) as previously described [41]. T-lymphocytes were double stained with anti-CD3 (A 0452; Dako) followed by anti-CD8 (1A5; Vector Laboratories), and BrdU+ cells were examined using anti-BrdU (Bu20A; Dako) as previously described [29]. Because the CD4 antigen is not optimally detected in routine paraffin embedded sections, we used double CD3 and CD8 to detect CD3+ cells and then determined if they were CD8+ or CD8−. CD3+CD3− cells in the CNS would include CD3+CD4+ lymphocytes. Productive SIV infection was determined with anti-SIV-p28 (3F7; Fitzgerald Industries International) and by in situ hybridization for SIV RNA using anti-digoxigenin labeled SIVmac239 antisense riboprobes that span the entire SIVmac genome (Lofstrand Labs) as previously described [64]. Hybridization specificity was confirmed in each experiment using the SIVmac239 sense probe and matched tissue from uninfected rhesus macaques. For quantification, at least 3 non-serial blind-coded sections from all tissues were stained for each marker. Tissue sections were examined with a Zeiss Axio Imager M1 microscope (Carl Zeiss MicroImaging, Inc.) using a Plan-Apochromat x20/0.8 Korr objective and analyzed by one unblinded and one blinded observer using Adobe Photoshop v11.0.2 software. The minimum number of arbitrary visual fields analyzed in each tissue was 24. From this number a median number of cells per tissue region was calculated. Data are represented as the number of positive cells per unit area (cells/mm^2^), and each point represents the mean number of positive cells in the three tissue regions examined from a single animal.

### Nucleic acid isolation and qPCR for SIV DNA loads in tissues

For each tissue examined, ten 15 µm frozen sections were homogenized and washed in 1× PBS (Ca^2+^/Mg^2+^ free; Mediatech Inc.) prior to genomic DNA isolation using the AllPrep DNA/RNA Mini Kit (Qiagen) according to manufacturers instructions. For each sample, 100 ng of gDNA was loaded in triplicate wells. The concentration of the gDNA was calculated using the Qubit 2.0 Fluorometer (Invitrogen). A standard curve was added to each PCR plate, consisting of a plasmid containing 1 copy of the SIV gag gene that was serially diluted from 1e9 copies down to 1 copy per microliter. Each quantitative PCR reaction contained 5 µl of a standard serial dilution or sample (diluted to 20 ng/µl) and 20 µl of reaction master mix containing 12.5 µl Invitrogen 2× TaqMan Universal Mastermix 2, 2.25 µl each of 10 uM forward and reverse primers, 0.625 µl of 10 µM TaqMan probe, and 2.375 µl of water. The forward and reverse primers ShehuF 5′-AATTAGATAGATTTGGATTAGCAGAAAGC and ShehuR 5′-CACCAGATGACGCAGACAGTATTAT and the MGB TaqMan probe ShehuP 6FAM-CAACAGGCTCAGAAAA-MGBNFQ were used as described previously [65]. The PCR was performed using Applied Biosystems 7500 Fast Real-Time PCR System under the following conditions: 95°C 10 min followed by 45 cycles of 94°C 15 s and 60°C 60 s. The lowest limit of detection of the assay was 50 copies per reaction. The number of viral gag gene DNA copies per 100 ng of total tissue gDNA was calculated using Applied Biosystems 750 Software v2.0.5.

### Statistical methods

Statistical analyses were conducted using Prism version 6.0 (GraphPad Software, Inc.). To detect significant changes in NAA/Cr metabolite ratios during disease progression, analysis of variance with repeated measures (RM-ANOVA) was used. If significant by RM-ANOVA (P<0.05), Holm-Šídák post-tests were used to isolate significant differences between time points and treatment groups. To determine whether the NAA/Cr slopes between treatment groups were significantly different, linear regression analyses were used to calculate r^2^ values for each treatment group and P values for differences in NAA/Cr slopes between groups in each brain region. All other P values were calculated using Student's two-tailed, unpaired t tests. Statistical significance was defined as P<0.05. Data are presented as the mean ± the standard error of the mean (SEM).

### Ethical treatment of animals

The treatment of animals was in accordance with the Guide for the Care and Use of Laboratory Animals of the Institute of Laboratory Animal Resources (8^th^ edition). The studies were performed with the approval of the Massachusetts General Hospital Subcommittee on Research and Animal Care, and the Institutional Animal Care and Use Committee of Harvard University. Animals were housed according to the standards of the American Association for Accreditation of Laboratory Animal Care. After infection with SIV, animals were individually housed, but received all other components of the NEPRC Environmental Enrichment Program. The Enrichment Program was supervised by NEPRC veterinarians in collaboration with Animal Behavioral staff, not by the PI. Enrichment was provided through manipulatable devices, food items, structural and environmental enhancements, and positive human interaction. Animals did not undergo food or water deprivation at any time during the study and were monitored daily for evidence of disease and changes in appetite and behavior. Clinical support was administered under the direction of an attending veterinarian and included antibiotics, analgesics, and intravenous fluids. Animals were anesthetized with ketamine-HCL and euthanized by intravenous pentobarbital overdose. The New England Primate Research Center (NEPRC) Protocol Number for this study is 04420 and the Animal Welfare Assurance Number is A3431-01.

## Supporting Information

Figure S1 **There is no difference between plasma and cerebrospinal fluid (CSF) viral loads in natalizumab treated and non-treated animals.** (A) Comparable levels of SIV RNA were seen in early untreated (n = 3, open circles) and natalizumab treated animals (n = 6, filled circles), with no effect of antibody administration on days 0, 7, and 14 post infection (dark grey arrows) on the high plasma viral loads visible by 8 dpi. Late natalizumab treatment on days 28, 34, and 41 post infection (light grey arrows) also did not affect plasma SIV RNA, with sustained concentrations of concentrations of virus in the plasma of late treated macaques (n = 4, filled squares) being even higher than that of untreated animals sacrificed with AIDS (n = 4, open squares). (B) Similar levels of SIV RNA were detected in the CSF of late untreated and natalizumab treated animals throughout infection. CSF samples from early untreated animals were not available, however concentrations of CSF SIV RNA in early natalizumab treated animals at 21 dpi were comparable to that of late untreated and natalizumab treated animals at 20 dpi.
(TIF)
