# Supplementary figures and images for: Anti-α4 Antibody Treatment Blocks Virus Traffic to the Brain and Gut Early, and Stabilizes CNS Injury Late in Infection
Source: PLoS Pathog. 2014 Dec 11;10(12):e1004533. doi: 10.1371/journal.ppat.1004533 (PMC4263764; doi:10.1371/journal.ppat.1004533)

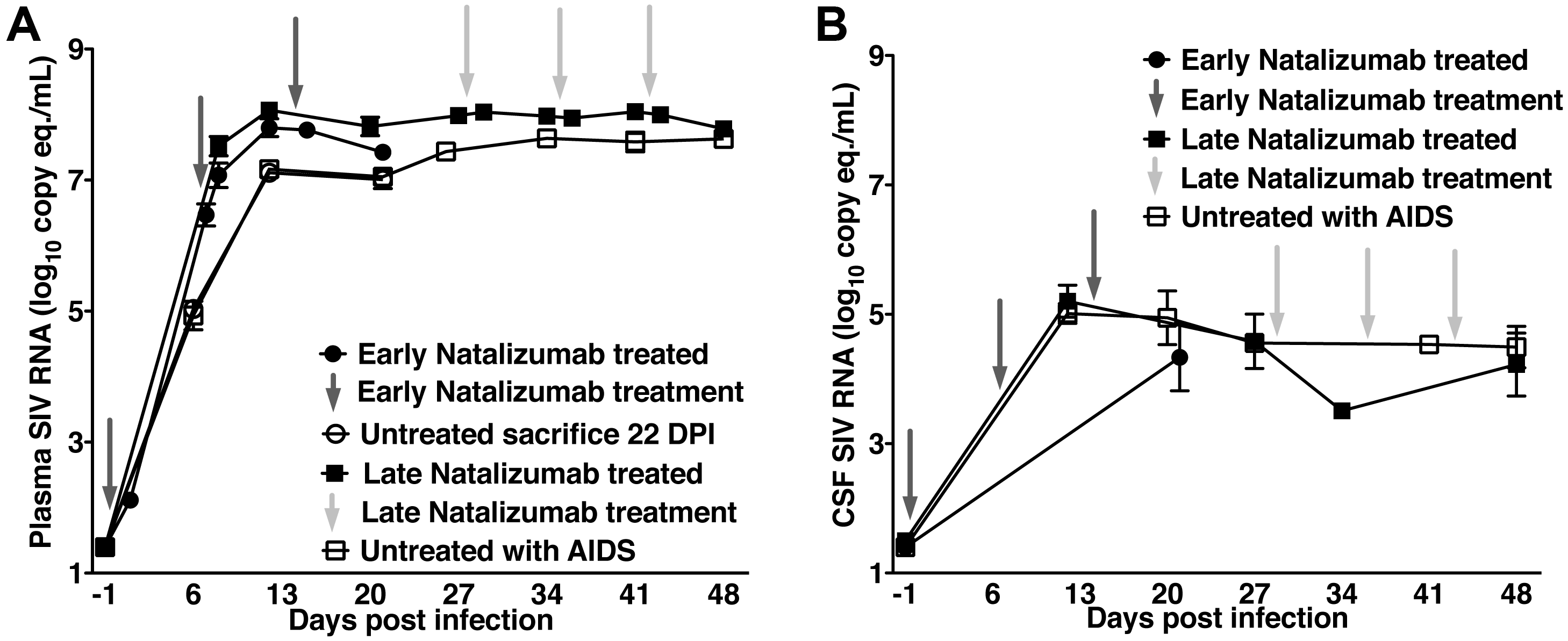

Supplement: Figure S1 — There is no difference between plasma and cerebrospinal fluid (CSF) viral loads in natalizumab treated and non-treated animals. (A) Comparable levels of SIV RNA were seen in early untreated (n = 3, open circles) and natalizumab treated animals (n = 6, filled circles), with no effect of antibody administration on days 0, 7, and 14 post infection (dark grey arrows) on the high plasma viral loads visible by 8 dpi. Late natalizumab treatment on days 28, 34, and 41 post infection (light grey arrows) also did not affect plasma SIV RNA, with sustained concentrations of concentrations of virus in the plasma of late treated macaques (n = 4, filled squares) being even higher than that of untreated animals sacrificed with AIDS (n = 4, open squares). (B) Similar levels of SIV RNA were detected in the CSF of late untreated and natalizumab treated animals throughout infection. CSF samples from early untreated animals were not available, however concentrations of CSF SIV RNA in early natalizumab treated animals at 21 dpi were comparable to that of late untreated and natalizumab treated animals at 20 dpi. (TIF) [file ppat.1004533.s001.tif]
